# Green rush and red warnings: Retrospective chart review of adverse events of interactions between cannabinoids and psychotropic drugs

**DOI:** 10.3389/fphar.2024.1500312

**Published:** 2024-10-22

**Authors:** Adrian Andrzej Chrobak, Jarosław Woroń, Marcin Siwek

**Affiliations:** ^1^ Department of Adult Psychiatry, Chair of Psychiatry, Jagiellonian University Medical College, Kraków, Poland; ^2^ Department of Clinical Pharmacology, Chair of Pharmacology, Faculty of Medicine, Jagiellonian University Medical College, Kraków, Poland; ^3^ Department of Anesthesiology and Intensive Care No. 1, Department of Internal Medicine and Geriatrics, University Hospital in Cracow, Kraków, Poland; ^4^ University Center for Monitoring and Research on Adverse Drug Effects in Krakow, Kraków, Poland; ^5^ Department of Affective Disorders, Chair of Psychiatry, Jagiellonian University Medical College, Kraków, Poland

**Keywords:** thc, cbd, antipsychotic drugs, sertraline, antidepressants, cytochrome, p-glycoprotein, medical marijuana

## Abstract

**Aim:**

Our objective was to systematically assess the prevalence and clinical features of adverse events related to interactions between cannabinoids and psychotropic drugs through a retrospective chart review.

**Methodology:**

1586 adverse event reports were assessed. Cases included in the analysis showed a high probability of a causal relationships between cannabinoid-psychotropic drug interactions and adverse events. Data extracted included age, sex, psychotropic drug, cannabinoid products, other medications, and the clinical outcomes and mechanisms of these interactions.

**Results:**

Cannabinoids were involved in 8% of adverse events associated with the concomitant use of psychotropic drugs and other preparations. We identified 20 reports in which side effects presented a causal relationship with the use of psychotropic drugs and cannabinoids. Preparations containing 18% or more tetrahydrocannabinol (THC), presented significant side effects with the following antidepressants: mianserine (restless legs syndrome, urogenital pain, ventricular tachycardia), mirtazapine (pancreatitis, hyperhidrosis, arthralgia), quetiapine (myocarditis, renal failure, bradycardia, sialorrhea), haloperidol (ventricular arrhythmia, prolonged QTc), aripiprazole (prolonged QTc), ventricular tachycardia) and cariprazine (stomach pain, hepatotoxicity), sertraline (ataxia, hyperactivity, coma, hallucinations, anxiety, agitation, tachycardia, panic attacks, disorientation, headache, dizziness, blurry vision, severe emesis, xerostomia, dry eyes), trazodone (disorientation, memory impairment, sedation), fluvoxamine (tachycardia, tachypnoea, dysarthria, auditory hallucinations). Two out of 20 reports (10%) analyzed in our study was related with the simultaneous use of cannabidiol (CBD) oil and sertraline. Concomitant use of those substances was associated with the adverse events in form of diarrhea, emesis, fever and severe fatigue.

**Conclusion:**

Clinicians need to closely monitor adverse events resulting from the combined use of cannabinoids and psychotropic medications. The accumulation of side effects and pharmacokinetic interactions (including CYP and p-glycoprotein inhibition) between these drugs can lead to clinically significant adverse outcomes.

## 1 Introduction

Cannabis sativa contains over 400 compounds, including more than 100 phytocannabinoids that influence human health and show medicinal promise (Rock and Parker, 2021). Among these, delta-9-tetrahydrocannabinol (THC) is the most prevalent cannabinoid, known for its intoxicating effects and therapeutic benefits in treating chemotherapy-induced nausea and vomiting, pain, and muscle spasticity (National Academies of Sciences, Engineering, and Medicine et al., 2017; Smith and Gruber, 2023). In contrast, cannabidiol (CBD), usually the second most abundant cannabinoid, is non-intoxicating and has been reported to have therapeutic effects for a variety of conditions such as seizure disorders, anxiety, pain, and inflammation (White, 2019). Moreover, growing number of clinical trials indicate that CBD presents antipsychotic properties that have an beneficial effects in patients with schizophrenia as well as in the people at clinical high risk for psychosis (Rohleder et al., 2016; McGuire et al., 2018; Leweke et al., 2021; Bhattacharyya et al., 2024). Over the past 10 years, there has been a significant increase in cannabis use, leading to more patients using it alongside their medications. This scenario poses potential issues since cannabinoids can act as either perpetrators or substrates when combined with other drugs, potentially causing adverse events and reduction of treatment effectiveness. Despite the growing popularity of cannabinoids, there are almost no studies analyzing the presence of significant drug-drug interactions (DDI) associated with the use of those substances in “real-world” patients population (Vázquez et al., 2020). Our previous research has shown that plant-based preparations are often a significant factor in clinically relevant interactions with psychotropic drugs and the resulting adverse events (Woroń and Siwek, 2018; Siwek et al., 2023). Therefore, we believe that the presence of drug-drug interactions (DDI) between cannabinoids and these medications could be a common phenomenon.

Polytherapy, which involves the concurrent use of two or more medications, is prevalent in clinical psychiatry. In the United States, up to one-third of patients are prescribed at least three psychotropic drugs, and this percentage is increasing over time (Mojtabai and Olfson, 2010). Using even two medications concurrently can pose risks of adverse interactions, and the likelihood of such interactions becomes certain with the simultaneous use of seven drugs (Vickers et al., 2006; McIntyre et al., 2016; Schatzberg and Nemeroff, 2017; Woroń and Siwek, 2018). This situation can lead to drug toxicity, a rise in adverse reactions, and notably, a significant risk of patient non-compliance (Kukreja et al., 2013). Polytherapy typically results in polypharmacy—a term that refers to the concurrent use of multiple medications, ranging from two to eleven depending on definitions (Masnoon et al., 2017). Such practices can lead to inadequate and insufficient medication use, often associated with a lack of the expected therapeutic efficacy (Woroń and Siwek, 2018).

Since cannabinoids are frequently used as adjunctive therapy, the likelihood of DDIs increases. Consequently, their therapeutic use may influence the metabolism of other drugs through inhibition or induction of cytochrome complexes and/or p-glycoprotein transporter, as well as through addition of side effects (summarized in Table 1). However, limited human studies investigating the DDIs of cannabinoids with other prescribed medications have been documented in literature (Geffrey et al., 2015; Gaston et al., 2017; Morrison et al., 2019; White, 2019; Vázquez et al., 2020), with some being merely case reports (Grayson et al., 2017; Leino et al., 2019; Madden et al., 2020). Given the frequent use of psychotropic drugs and cannabinoids, DDI between those two groups of medicines should be a common phenomenon. The aim of our study is to evaluate the incidence and characteristics of adverse interactions of psychotropic drugs and cannabinoids in a retrospective chart review.

**TABLE 1:** Common side effects and possible interaction mechanisms of the analyzed cannabinoids. THC–Tetrahydrocannabinol, CBD–Cannabidiol, CYP–Cytochromes P450, ↑ induction and ↓ inhibition (−) – no action.

	Side effects	Cytochromes involved in cannabinoid metabolism	Interactions with cytochrome	Interactions with p-glycoprotein
THC	Gastrointestinal symptoms, drowsiness, slurred speech, blurred vision, mental clouding, confusion, disorientation, gastrointestinal symptoms, headache, dysphoria, euphoria, hallucinations, paranoia, dry mouth, hypotension (Deshpande et al., 2015; Ng and Keshock, 2023; Pressman et al., 2024)	CYP2C9 CYP2C19 CYP3A4 (Kocis and Vrana, 2020)	↓ CYP3A4 ↓ CYP2B6 ↓↑ CYP2C9 ↓ CYP2C19 ↓ CYP2D6 ↓ CYP2E1 ↓ CYP2J2 ↑ CYP1A1 (Qian et al., 2019; Bansal et al., 2020; Doohan et al., 2021; Nasrin et al., 2021; Bardhi et al., 2022)	(−) Chen et al. (2021)
CBD	gastrointestinal symptoms, somnolence, loss of appetite, and hypertransaminasemia (ALT/AST), seizures, rash. (Souza et al., 2022)	CYP2C9 CYP2C19 CYP3A4 CYP2C8 CYP1A2 CYP2A6 (Kocis and Vrana, 2020)	↓CYP2C9 ↓CYP2C19 ↓ CYP2D6 ↓↑ CYP3A4 ↓↑ CYP1A2 ↓↑ CYP2B6 ↓CYP2C8 Zendulka et al. (2016); Kocis and Vrana (2020); Balachandran et al. (2021); Beers et al. (2021)	↓ Zhu et al. (2006)

## 2 Materials and methods

Prevalence and clinical characteristic of adverse events associated with the drug-drug interactions between cannabinoids and psychotropic drugs were evaluated in retrospective chart review. Study has been performed in accordance with the methodology of our previous analyses of psychotropic drug interactions (Woroń and Siwek, 2018; Woroń et al., 2019; Siwek et al., 2020; 2023). Retrospective chart review was performed by all of the authors. The dataset involved reports on the occurrence of adverse events resulting from drug-drug interactions. They were evaluated at the University Centre for Monitoring and Research on Adverse Drug Effects, Department of Clinical Pharmacology at Jagiellonian University Medical College in Cracow. This unit is authorised by Polish legal acts to monitor and report complications associated with pharmacotherapy, as well as to provide specialist consultations of clinics and hospitals from Holy Cross, Lesser Poland, Silesian and Subcarpathian regions. The Centre cooperates with Department of Affective Disorders of the Jagiellonian University Medical College due to the increasing number of adverse events associated with the use of psychotropic drugs. Annually, this unit performs approximately 850–1100 consultation.

In this study we have analysed reports of adverse events that came from all over the Poland in the period between 01.01.2023-31.12.2023. They were evaluated when the following criteria have been met: 1) patient used at least one psychotropic drug, 2) patients used at least one preparation containing cannabinoids (CBD or THC), 3) high probability of a causal relationship in terms of pharmacokinetic, pharmacodynamic interactions or the interactions associated with the aggregation of the side effects caused by the simultaneous use of cannabinoids and psychotropic drugs has been established in the pharmacoepidemiological analysis.

**FIGURES 1:**
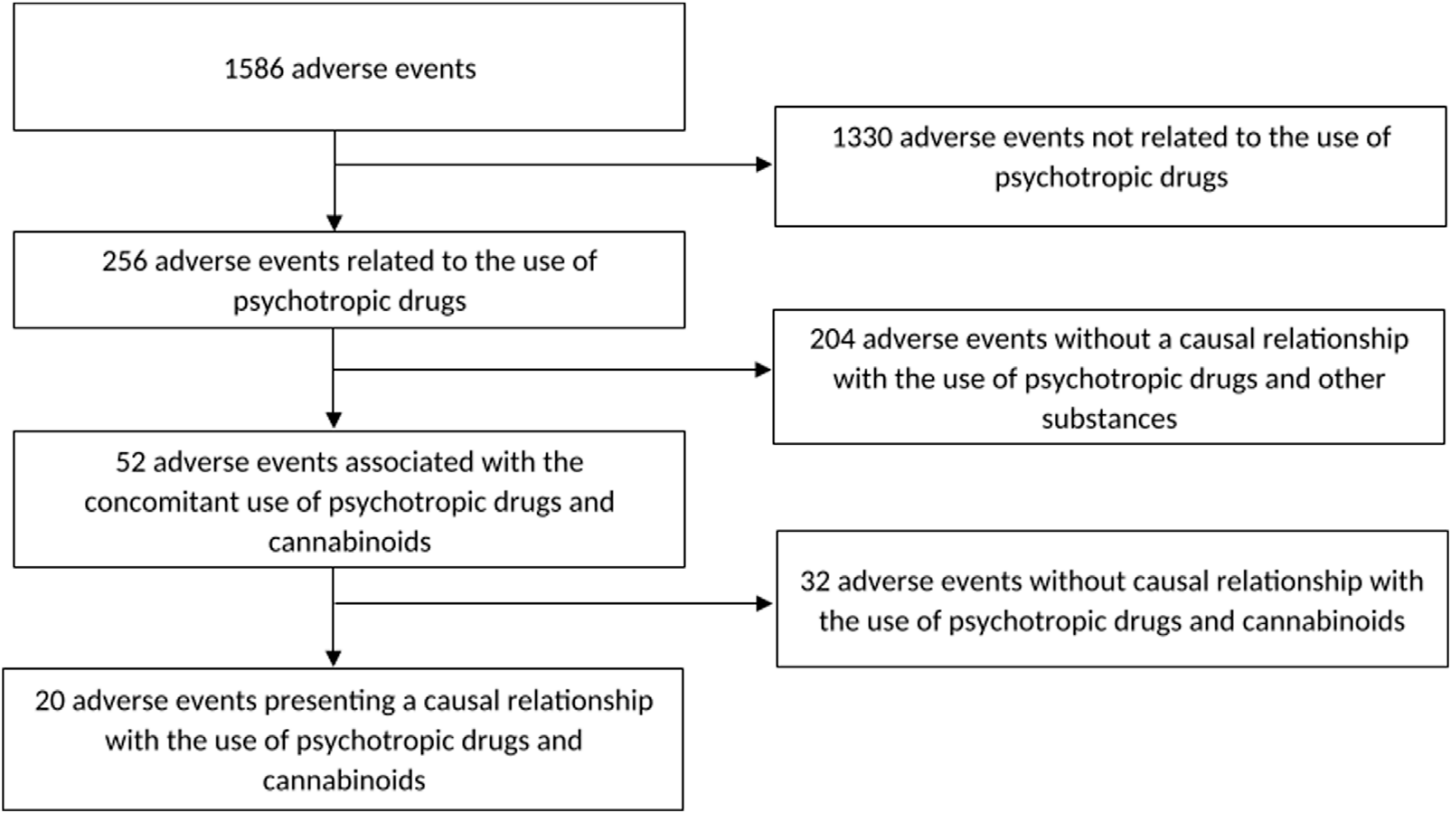
Flow chart of the retrospective chart review.

Figure 1 shows a flow chart of our retrospective chart review. 1586 reports of adverse events have been evaluated. 256 cases presented causal relationship with the use of psychotropic drugs from which 52 cases were associated with the concomitant use of preparations containing cannabinoids. Among those, 20 cases presented high probability of a causal relationship between psychotropic drugs and cannabinoids interaction and the occurrence of side effects. Thus, cannabinoids were involved in 8% (20/256) of adverse events associated with the concomitant use of psychotropic drugs and other preparations.

## 3 Results

Table 2 demonstrates data extracted from 20 reports of adverse events that presented a causal relationship with the use of preparations containing cannabinoids and psychotropic drugs. They were thirteen women and seven men. The mean age of the group was 53.7 ± 13. Most of the adverse events were associated with the use of antidepressants (14 patients, 70%), particularly sertraline (seven cases, 35%), mianserine (three patients, 15%), mirtazapine (two patients, 10%), fluvoxamine (one patient, 5%), trazodone (one patient, 5%). Six cases (30%) involved the use of antipsychotic drugs, namely, quetiapine (two patients, 10%), aripiprazole (one patient, 5%), cariprazine (one patient, 5%), haloperidol (one patient, 5%). When it comes to preparations containing cannabinoids, the majority of adverse events were associated with the use medical marijuana with approximate 18%–22% of THC and 1% of CBD (18 patients, 90%). Two adverse events were related to the use of 10% CBD oil (10% of cases). There were no reports of side effects associated with concomitant use of antidepressants and FDA/EMA approved CBD and CBD/THC formulations such as Sativex or Epidiolex. In all of the cases cannabinoids were used for the treatment of pain, particularly associated with cancer (nine patients, 45%), endometriosis (four patients, 20%), osteoarthritis (two patients, 10%) visceral hyperalgesia (one patient, 5%), chronic pancreatitis (one patient, 5%), prostatodynia (one case, 5%), postherpetic neuralgia (one patient, 5%), fibromyalgia (one patient, 5%). Pharmacokinetic interactions have been shown as the leading mechanism of adverse event in every report. In case of one patient presence of the addition of side effects has been shown. Detailed description of the proposed mechanisms of interactions and their clinical consequences has been demonstrated in Table 1.

**TABLE 2:** Interactions between cannabinoids and cananbinoids in the analyzed group and possible interaction mechanisms. THC–Tetrahydrocannabinol, CBD–Cannabidiol, CYP–Cytochromes P450.

Plant products containing cannabinoids and its composition	Indication for the use of cannabinoid	Psychotropic drug and its dose	Sex/age	Other concomitant medications	Clinical consequences of the interaction	Possible interaction mechanism
Cannabis flos Aurora Deutschland GMBH 22/1 (Ghost Train Haze), THC 22%; CBD <1%	Cancer pain (lung cancer)	Mianserine 90 mg/day	M/51	Oxycodone, meloxicam, gabapentine, intranasal fentanyl	Restless legs syndrome, urogenital pain	Pharmacokinetic: inhibition of CYP3A4 by cannabinoids increased concentration and side effects of mianserine (metabolized by CYP3A4)
	Cancer pain	Mianserine 90 mg/day	M/56	Morphine, pregabalin, dexketoprofen	Restless legs syndrome	Pharmacokinetic: inhibition of CYP3A4 by cannabinoids increased concentration and side effects of mianserine (metabolized by CYP3A4)
*Cannabis flos Aurora 22/1 (Delahaze), THC 22%, CBD 1%*	Cancer pain	Sertraline 100 mg/day	M/41	Oxycodone, metamizole, ketoprofen, zoledronic acid	Ataxia, initial hyperactivity then coma. Hospitalization in intensive care unit	Pharmacokinetic: inhibition of CYP3A4 and CYP2C9 by sertraline increased concentration and side effects of cannabinoids (metabolized by CYP3A4 and CYP2C9) Pharmacokinetic: inhibition of CYP2C19 by cannabinoids increased concentration and side effects of sertraline (metabolized by CYP2C19)
	Cancer pain (vulvar cancer)	Mirtazapine 45 mg/day	F/78	Oxycodone, gabapentin, dexketoprofen	Pancreatitis, hyperhidrosis	Pharmacokinetic: inhibition of CYP3A4 by cannabinoids increased concentration and side effects of mirtazapine (metabolized by CYP3A4)
*THC 20% CBD 1%*	Visceral hyperalgesia	Quetiapine 150 mg/day	F/67	Metamizole, drotaverine	Myocarditis	Pharmacokinetic: inhibition of CYP3A4 by cannabinoids increased concentration and side effects of quetiapine (metabolized by CYP3A4)
	Pain caused by chronic pancreatitis	Quetiapine 100 mg/day	F/36	Oxycodone, drotaverine, pancreatin, trimebutine, gabapentin	Acute noninflammatory renal failure 5 days after introduction of cannabinoids	Pharmacokinetic: inhibition of CYP3A4 by cannabinoids increased concentration and side effects of quetiapine (metabolized by CYP3A4)
	Cancer pain	Sertraline 100 mg/day	F/71	Oxycodone/naloxone, pregabalin, metamizole, dexketoprofen, dapagliflozin	Auditory and visual hallucinations, anxiety, agitation, tachycardia (160 bpm)	Pharmacokinetic: inhibition of CYP3A4 and CYP2C19 by sertraline increased concentration and side effects of THC (metabolized by CYP3A4 and CYP2C9) Pharmacokinetic: inhibition of CYP2C19 by cannabinoids increased concentration and side effects of sertraline (metabolized by CYP2C19)
	Pain caused by osteoarthritis	Cariprazine	F/65	Chondroitin sulfate, etoricoxib	Stomach pain, hepatotoxicity (ALT = 850 U/L, GGTP = 480 U/L)	Pharmacokinetic: inhibition of CYP3A4 by cannabinoids increased concentration and side effects of cariprazine (metabolized by CYP3A4)
*Cannabis flos Aurora 20/1 (Pink Kush), THC 20% CBD 1%*	Fibromyalgia	Fluvoxamine 100 mg/day	F/46	Pregabalin, pitavastatin, ezetimibe	Tachycardia (170 bpm), tachypnoea, dysarthria, auditory hallucinations	Pharmacokinetic: inhibition of CYP3A4 by fluvoxamine increased concentration and side effects of THC (metabolized by CYP3A4)
	Cancer pain	Sertraline 150 mg/day	F/61	Oxycodone, gabapentin, paracetamol, metamizole	Ventricular tachycardia, panic attacks, disorientation	Pharmacokinetic: inhibition of CYP3A4 and CYP2C19 by sertraline increased concentration and side effects of THC (metabolized by CYP3A4 and CYP2C9)
*Cannabis sativa L, Canopy Growth 19, (Lemon Skunk, Red No 2), THC 19% (* ± *10%), CBD < 1%*	Cancer pain	Sertraline 100 mg/day	M/54	Morphine, ketoprofen, pregabalin, lactulose	Disorientation, headache, dizziness, blurry vision	Pharmacokinetic: inhibition of CYP3A4 and CYP2C19 by sertraline increased concentration and side effects of THC (metabolized by CYP3A4 and CYP2C9) Pharmacokinetic: inhibition of CYP2C19 by cannabinoids increased concentration and side effects of sertraline (metabolized by CYP2C19)
	Cancer pain	Quetiapine 200 mg/day	F/58	Morphine, ketoprofen, metamizole, pregabalin, lidocaine iv	Bradycardia (28 bpm), sialorrhea	Pharmacokinetic: inhibition of CYP3A4 by cannabinoids increased concentration and side effects of quetiapine (metabolized by CYP3A4)
	Prostate pain	Trazodone 300 mg/day	M/58	Tamsulosin, gabapentin, metamizole	Disorientation, memory impairment, sedation	Pharmacokinetic: inhibition of CYP3A4 by trazodone increased concentration and side effects of THC (metabolized by CYP3A4)
	Cancer pain	Haloperidol 3 mg/day	M/55	Morphine, duloxetine, ketoprofen, pantoprazole	Ventricular arrythmia, prolonged QTc (540 m)	Pharmacokinetic: inhibition of CYP3A4 by cannabinoids increased concentration and side effects of haloperidol (metabolized by CYP3A4)
*Cannabis Flos THC 18%, CBD ≤ 1%*	Pain caused by endometriosis	Sertraline 150 mg/day	F/38	Goserelin, ketoprofen, metamizole	Severe emesis that required hospitalization, xerostomia, dry eyes	Pharmacokinetic: inhibition of CYP3A4 and CYP2C19 by sertraline increased concentration and side effects of THC (metabolized by CYP3A4 and CYP2C9) Pharmacokinetic: inhibition of CYP2C19 by cannabinoids increased concentration and side effects of sertraline (metabolized by CYP2C19)
	Pain caused by endometriosis	Mianserine 60 mg/day	F/26	Levonorgestrel intravaginal contraception, oxycodone, ketoprofen	Ventricular tachycardia	Pharmacokinetic: inhibition of CYP3A4 by cannabinoids increased concentration and side effects of mianserine (metabolized by CYP3A4)
	Pain caused by endometriosis	Mirtazapine 30 mg/day	F/44	Oxycodone, pregabalin	Arthralgia (NRS = 6-8/10)	Pharmacokinetic: inhibition of CYP3A4 by cannabinoids increased concentration and side effects of mirtazapine (metabolized by CYP3A4)
*Cannabis flos Tilray, THC 18%, CBD 1%*	Postherpatic neuralgia	Aripiprazole 30 mg/day	M/55	Transdermal lidocaine, pregabalin	Prolonged QTc (560 m), ventricular tachycardia	Pharmacokinetic: inhibition of CYP3A4 by cannabinoids increased concentration and side effects of aripiprazole (metabolized by CYP3A4)
*CBD oil (10%)*	Pain caused by osteoarthritis	Sertraline 100 mg/day	F/68	Lornoxicam, oxycodone, topical diclofenac	Diarrhea, emesis, fever	Pharmacokinetic: inhibition of CYP3A4 and CYP2C19 by sertraline increased concentration and side effects of CBD (metabolized by CYP3A4 and CYP2C9) Pharmacokinetic: inhibition of CYP2C19 and CYP2C9 and p-glycoprotein by CBD increased concentration of sertraline (metabolised by CYP2C19 and CYP2C9 and transported by p-glycoprotein) Addition of side effects: CBD may induce gastrointestinal symptoms, including diarrhoea 20% of patients treated with sertraline present diarrhoea
	Pain caused by endometriosis	Sertraline 100 mg/day	F/47	Ethinil estradiol/gestoden, ketoprofen, tramadol	Severe fatigue that prevents daily activities	Pharmacokinetic: inhibition of CYP3A4 and CYP2C19 by sertraline increased concentration and side effects of CBD (metabolized by CYP3A4 and CYP2C9) Pharmacokinetic: inhibition of CYP2C19 and CYP2C9 and p-glycoprotein by CBD increased concentration of sertraline (metabolised by CYP2C19 and CYP2C9 and transported by p-glycoprotein)

## 4 Discussion

To our best knowledge, this is the first retrospective chart review that demonstrates prevalence and clinical presentation of the side effects caused by the simultaneous use of cannabinoids and psychotropic drugs. Thorough analysis of 257 reports indicated that 8% of adverse events caused by interactions of psychotropic drugs with other substances were the result of their simultaneous use with cannabinoids.

In case of 11 out of 18 (61%%) reports related to the use of THC, adverse effects were associated with the inhibition of CYP3A4, CYP2D6, CYP2C19 and CYP2A1 by cannabinoids, what lead to the increased concentration and severity of side effects of drugs metabolised by those protein complexes. We have identified cases of side effects associated with the use of marijuana preparations containing 18% or more THC, and psychotropic drugs, particularly mianserine (restless legs syndrome, urogenital pain, ventricular tachycardia), mirtazapine (pancreatitis, hyperhidrosis, arthralgia (NRS = 6-8/10)), quetiapine (myocarditis, acute non-inflammatory renal failure, bradycardia (28 bpm), sialorrhea), haloperidol (ventricular arrhythmia, prolonged QTc (540 m)), aripiprazole (prolonged QTc (560 m), ventricular tachycardia) and cariprazine (stomach pain, hepatotoxicity (ALT = 850 U/L, GGTP = 480 U/L)).

In seven of out 18 cases (39%) related to the simultaneous use of THC preparations and psychotropic drugs, main mechanism of adverse events was related to the inhibition of CYP3A4 and CYPC19 involved in metabolism of this cannabinoid. Concomitant use of THC and following antidepressant drugs was related with the increased concentration and the severity of side effects of cannabinoids: sertraline (ataxia, hyperactivity, coma, auditory and visual hallucinations, anxiety, agitation, tachycardia, panic attacks, disorientation, headache, dizziness, blurry vision, severe emesis that required hospitalization, xerostomia, dry eyes), trazodone (disorientation, memory impairment, sedation), fluvoxamine (tachycardia (170 bpm), tachypnoea, dysarthria, auditory hallucinations).

Abovementioned adverse events related to interactions between antidepressants and cannabinoids support the predictions of significant pharmacokinetic DDIs between those substances that came from *in vitro* and animal studies (Foglia et al., 2017). To our best knowledge there are no clinical studies indicating the presence of side effects associated with interactions between cannabinoids and mirtazapine, mianserin, fluvoxamine and trazodone. Given the fact that the use of trazodone and mirtazapine has been proposed in the treatment of cannabis use disorders (Sherman and McRae-Clark, 2016; Mazza et al., 2022), clinicians should be aware of the risk associated with the interactions that may emerge during the relapse of cannabis abuse.

In our research, sertraline emerged as the drug most associated with adverse events when used with cannabinoids. The frequent occurrence of these reactions might be attributed to the bidirectional interactions between the substances. Specifically, sertraline disturbs the metabolism of cannabinoids by inhibiting the CYP3A4 and CYP2C9, while THC inhibits the CYP2C19 cytochrome, thereby increasing the concentration of sertraline (Zendulka et al., 2016; Kocis and Vrana, 2020; Doohan et al., 2021; Nasrin et al., 2021; Vaughn et al., 2021). The occurrence of clinically significant side effects in this group aligns with recent pharmacokinetic models indicating that the use of THC and/or CBD elevates sertraline levels. Additionally, the combined administration of CBD and SSRIs metabolised by CYP2C19 increases the risk of diarrhoea, dizziness, fatigue, and cough (Vaughn et al., 2021).

There is scarce data evaluating interactions between cannabinoids and antipsychotic drugs. In our best knowledge there are no reports of DDI associated with the use of marijuana and cariprazine or aripiprazole. Pham et al., 2021 presented a case of patient that smoked marijuana mixed with crushed haloperidol tablets, what resulted in suicidal ideation, psychosis and acute dystonia. Authors suggested that one of the mechanisms of this side event involves increased concentration of haloperidol via inhibition of CYP3A4 leading to the development of extrapyramidal symptoms (Pham et al., 2021). Systematic review and meta-analysis of Foglia et al., 2017 have shown that cannabis use significantly increases the risk of non-adherence to antipsychotic medication. Given the fact that the side effects of psychopharmacotherapy are significant cause of patients non-compliance, it is likely that symptoms caused by interactions between cannabinoids and antipsychotic drugs may contribute to non-adherence observed in this clinical group (Foglia et al., 2017). To our best knowledge the occurrence and impact of side events in group of patients concomitantly using abovementioned substances has not been evaluated. Further studies are required to address this issue.

Two out of 20 reports (10%) analyzed in our study was related with the simultaneous use of CBD oil and sertraline. Concomitant use of those substances was associated with the adverse events in form of diarrhea, emesis, fever and severe fatigue. The occurrence of these side effects may result from complex interactions that involves: 1) inhibition of CYP2C19 and CYP3A4 by sertraline, leading to the increased concentration of CBD (Nemeroff et al., 1996); 2) inhibition of CYP2C19, CYP2C9 and p-glycoprotein by CBD leading to increased concentration of sertraline (Zhu et al., 2006; Zendulka et al., 2016; Kocis and Vrana, 2020; Balachandran et al., 2021; Beers et al., 2021); 3) addition of side effects in form of gastrointestinal symptoms (Souza et al., 2022; Wang et al., 2022). To our best knowledge there is only one case study evaluating interactions between CBD and sertraline. Nanan et al. (2022) have shown that simultaneous use of those drugs resulted in hyponatremic cognitive dysfunction in an intermediate CYP2C19 metabolized patient. Our results stay in line with the predictions based on area under the concentration-time curve rations (AUCR) indicating strong drug interactions with CBD mediated by CYP2C9 and CYP2C19 (Bansal et al., 2020). Clinical studies support the role of proposed mechanisms, indicating that oral administration of CBD leads to the increase of concentration of CYP2C19 substrates such as stripentol and hexobarbital (Benowitz et al., 1980; Morrison et al., 2019). Our results indicate that physicians should be aware of the risk of DDI associated with the use of CBD and antidepressant drugs metabolized by those cytochrome complexes.

In alignment with our recent study, the mean age of patients (54 ± 13) suggests that the higher risk of drug-drug interactions (DDIs) is likely age-related (Woroń and Siwek, 2018; Woroń et al., 2019; 2022; Siwek et al., 2023). It has been shown that older users are more inclined to view cannabis as safe and to use it, largely due to their earlier exposure to the substance (Abuhasira et al., 2019; Ellis et al., 2019). Also, this group often faces polypharmacy, increasing the DDI risk. Data showed 81% of older adults took at least one prescribed medication, 29% used five or more drugs, and 42% used at least one OTC medicine (Albert et al., 2014; Qato et al., 2016). Since CBD products are mostly sold without prescription, the low detection rate of interactions in our study is likely due to lack of awareness rather than infrequent adverse events.

## 5 Conclusion

- An evaluation of the pharmacokinetic profile of a patient’s medications is vital to identify any overlaps in cytochrome P450 isoenzymes involved in drug metabolism, which may influence their concentrations.
- Special attention should be given when psychotropic drugs are administered at maximum dosages or rapidly titrated, as pharmacokinetic interactions with cannabinoids could lead to surpassing therapeutic levels.
- The interaction between psychotropic drugs and cannabinoids can result in life-threatening adverse effects, such as myocarditis or renal failure.
- Patients should be questioned by both physicians (primary care or psychiatric) and pharmacists about OTC products containing cannabinoids and informed of the potential side effects when used concurrently with psychotropic drugs.
- The use of cannabinoids by the patient should be documented in their medical records to facilitate the monitoring of any adverse events related to these substances.

## Data Availability

The original contributions presented in the study are included in the article/supplementary material, further inquiries can be directed to the corresponding author.
